# Supplementary material for: Clinical and Functional Characteristics of the E92K CFTR Gene Variant in the Russian and Turkish Population of People with Cystic Fibrosis
Source: Int J Mol Sci. 2023 Mar 28;24(7):6351. doi: 10.3390/ijms24076351 (PMC10093870; doi:10.3390/ijms24076351)
Supplement: Supplementary file 1 [file ijms-24-06351-s001.zip › ijms-2195175-supplementary.pdf]

Table S1. Characteristics of patients with CF with an intestinal biopsy performed for the ICM method and a FIS assay on intestinal organoids.

Groups	Number of patients, age, gender	Name of the second variant	Nature of the violation	Clinical significance
E92K/E92K Group 1	3 ind., 2 m:1 w, 5, 8 and 12 y.o.	E92K	severe	pathogenic
E92K+class I: E92K /2143delT, E92K/CFTRdel1-11 Group 2	2 ind., 1m:1w, 7 and 5 y.o.	2143delT	severe	pathogenic
		CFTR del1-11	severe	pathogenic
E92K+ F508del Group 3	3 ind., 1m:2w, 3,9 and 11 y.o	F508del	severe	pathogenic
E92K+ class IV-V: E92K/L138ins, E92K/3849+10kbC→T Group 4	2 ind., 1m:1w, 2 and 13 y.o	L138ins	mild	pathogenic
		3849+10kbC→T	mild	pathogenic
F508del/F508del Group V	3 ind, 1m:2w, 12, 12 and 16 y.o	F508del	severe	pathogenic
wt/wt control group	13 ind /2 ind*	-	no violations	healthy

* Note: 13 people were examined using the ICM method and 2 using FIS assay on the intestinal organoids.

Table S2. The AUC data for FIS measurements

	0,128 μM forskolin							
	forskolin		forskolin + VX-770		forskolin + VX-809		forskolin + VX-770 +VX-809	
	mean	sd	mean	sd	mean	sd	mean	sd
E92K/L138ins	21,79	136,55	818,76	177,62	2181,67	263,00	3416,45	373,74
E92K/3849+10kbC-->T	-257,32	56,16	329,13	116,56	21,19	68,99	2312,50	71,35
E92K/F508del patient 3	132,06	135,41	-156,10	205,17	374,39	242,89	2588,68	607,58
E92K/F508del patient 2	-20,34	32,69	270,66	11,27	2630,92	54,74	3416,92	49,02
E92K/F508del patient 1	-161,27	40,88	89,74	14,01	1540,21	33,11	3299,00	32,08
E92K/ CFTRdel 1-11	-75,30	85,92	-144,43	50,92	-73,28	77,08	1373,67	25,54
E92K/2143delT	-78,58	80,05	-58,33	77,09	774,67	48,64	2588,28	36,32
E92K/E92K patient 3	-214,36	157,36	136,04	33,99	1928,41	23,91	3754,41	37,16
E92K/E92K patient 2	2,30	54,58	45,47	105,11	2002,80	52,97	2336,47	42,52
E92K/E92K patient 1	-162,66	166,74	32,27	115,65	1648,12	30,06	3838,65	24,72
	5 μM forskolin							
E92K/L138ins	1938,22	298,32	2492,72	108,29	5041,99	609,65	4136,74	574,48

E92K/3849+10kbC-->T	468,36	143,24	1142,03	220,99	2971,89	131,80	3226,94	88,38
E92K/F508del patient 3	236,20	66,20	469,63	144,00	5018,25	577,47	5515,72	699,85
E92K/F508del patient 2	-46,84	73,53	523,50	15,60	3756,37	95,05	4165,95	27,57
E92K/F508del patient 1	-174,99	95,52	87,99	59,24	2418,96	20,23	3855,71	74,39
E92K/ CFTRdel 1-11	-56,51	-56,51	52,62	48,80	1921,20	43,51	3127,24	68,93
E92K/2143delT	-347,17	109,34	-7,34	44,73	2261,42	63,37	3343,75	48,20
E92K/E92K patient 3	-25,99	125,94	212,95	28,58	3384,59	39,10	4469,15	74,71
E92K/E92K patient 2	-154,63	244,65	884,24	24,59	2455,45	71,80	3681,70	16,23
E92K/E92K patient 1	151,82	51,93	504,89	16,08	3531,16	33,39	4111,87	32,06
F508del/F508del	-67,98	187,85	448,59	169,24	647,68	325,01	1928,91	126,50
